# Urban gardening and neglected and underutilized species in Salvador, Bahia, Brazil

**DOI:** 10.1186/s13002-020-00421-0

**Published:** 2020-10-29

**Authors:** Manuela Alves da Cunha, Lidice Almeida Arlego Paraguassú, José Geraldo de Aquino Assis, Arthur Benjamin de Paula Carvalho Silva, Ryzia de Cassia Vieira Cardoso

**Affiliations:** 1 Escola de Nutrição, Universidade Federal da Bahia, Basílio Gama Street, Canela Campus, Salvador, Bahia 40110-907 Brazil; 2 Instituto Federal de Educação, Ciência e Tecnologia da Bahia, Emídio dos Santos Street, Barbalho Campus, Salvador, Bahia 40301-015 Brazil; 3 Instituto de Biologia, Universidade Federal da Bahia, 668, Barão de Jeremoabo Street, Ondina Campus, Salvador, Bahia 40170-115 Brazil; 4 Faculdades Integradas São Pedro – FAESA, 2220, Vitória Avenue, Monte Belo, Vitória, Espírito Santo, 29053-360 Brazil; 5 Escola de Nutrição, Universidade Federal da Bahia, Basílio Gama Street, Canela Campus, Salvador, Bahia 40110-907 Brazil

**Keywords:** Food systems, Urban agriculture, Genetic heritage, Food and nutrition security

## Abstract

**Background:**

Urban agriculture has been evidenced as a food production and environmental sustainability strategy, although it faces many obstacles in Latin American countries. Additionally, in urban areas, low consumption of fruit and greenery is noticeable, along with loss in food diversity, including the neglected and underutilized species (NUS), which involve potential to strengthen local food systems. For this reason, this work has sought to map urban gardens in the city of Salvador, Bahia, Brazil, characterizing their gardeners, and to systematize information regarding food produced and the use of NUS.

**Methods:**

The municipality’s urban gardens were mapped and data was collected from the gardeners. The study included two steps: (i) garden localization; (ii) on-site visits for interviews with gardeners and verification of cultivated food, destination of production, availability, and use of NUS.

**Results:**

Eighteen active food gardens were located, seventeen of which participated in the study: eight (8) communal (UCG) and nine (9) private (UPG). Respondents were on average 55.76 years old, mostly (52.9%) male, working at UPG (88.9%). Women predominated in the UCG (87.5%), with higher levels of education. For 52.9% of the interviewees, the garden was their main source of income. Food produced at the urban gardens was consumed by 82.4% of the gardeners and their families. In 70.6% of the gardens, production was also sold, while 47.1% donated. During the survey, 59 NUS were found and 76.5% of respondents reported consuming 19 of the species. NUS leaves, fruits, and seeds were found to be eaten raw, boiled, or sautéed in various preparations, especially *Coleus amboinicus* Lour. (76.5%), *Eryngium foetidum* L. (35.3%), *Talinum fruticosum* (L.) Juss., and *Pereskia aculeata* Mill (both 29.4%). Occurrence and utilization of NUS did not present significant associations with the gardens or gardeners (*p* > 0.05).

**Conclusions:**

Salvador urban gardens, even in small numbers and without government support, have produced affordable food for the local population, preserved food diversity, and the tradition of NUS cultivation and use. Thus, urban gardens are reaffirmed as relevant spaces that should be included in public policies in order to promote food and nutritional security, biodiversity, and urban environmental sustainability.

## Background

According to the United Nations, over the next 30 years, the world population is projected to grow by 2 billion people—from the current 7.7 to 9.7 billion in the year 2050 [1]. With increasing urbanization, decreasing arable land, and climate change, it is estimated that agriculture will face major challenges in the future. In addition, by 2030, 70% of the world population is projected to live in cities. Thus, urban agriculture emerges as an alternative to increasing global food production [2].

Worldwide, research has shown that food production through urban agriculture is increasing, with at least 100 million people involved and potential output reaching 50 kg/m^2^ annually [2–4]. However, in Latin America and Caribbean cities, this activity has not achieved its full potential and requires greater support from national, unit, and city governments [5, 6].

In Brazil, urban agriculture has shown slow development throughout its history, without a specific policy for the promotion and regulation of the activity [7, 8]. Across the country, urban agriculture initiatives have encountered numerous challenges, including lack of specific legal and policy frameworks, insufficient financial support and technical assistance, difficulties in accessing water of adequate quality, urban land-use restrictions, and lack of legal ownership of the spaces [9].

Parallelly, it is a fact that urbanization and industrialization have brought about several changes in the population’s demographic profile, quality of life, and food supply, with consequences on food and nutrition security (FNS) and on the health of individuals [10]. The previously vegetable-based diet has been replaced by high-energy-density alternatives, with soaring levels of sugars and fats, which can cause deleterious effects on human health [11].

In this scenario, the tradition of consuming plant species that have food potential but are not organized in a production chain has been increasingly forgotten [12–14]. These vegetables, referred to in Brazil as *Plantas Alimentícias Não Convencionais* (PANC), are internationally referred to as neglected and underutilized species (NUS) or *Especies Olvidadas y Subutilizadas*, in Spanish. The terms refer to those species of wild or semi-domesticated plants that are adapted to particular and often local environments that do not receive much attention and are often ignored by researchers, farmers, and lawmakers [15–18].

Neglected and underutilized crops help increase diversification of production and consumption of plant species while providing economic and environmental benefits as farmers can integrate them into crop rotation systems or plant them among other crops, protecting agrobiodiversity [19]. In Brazil, a wide variety of underutilized species may be present in urban flower beds or urban gardens, making contributions from an ecological, economic, nutritional, and cultural perspective. However, many of them suffer pejorative characterization, being classified as “weeds” since they appear in places where they were not cultivated [20].

In the Northeastern Brazilian city of Salvador, Bahia state, urban agriculture is characterized by the presence of urban gardens, which were first recorded during the foundation period of the city, in 1549. The activity continues to the present day and is considered a relevant strategy for local food security, as it provides a larger food supply and encourages the implementation of agroecological practices, in a scenario marked by social inequalities and pockets of poverty. In large part, the activity is developed spontaneously and confronted in its operation, without government support [21–23]. In this sense, research on urban gardens is still insufficient, and there are no systematizations about NUS in these environments.

It should be noted that studies on NUS in Brazilian urban agriculture are still scarce. Therefore, works in this area can contribute to the conservation and valorization of native species, as well as promoting sustainability and healthier eating practices, subsidizing public policies aligned with the food and nutrition security guidelines. Therefore, this research aimed to map urban gardens in the city of Salvador, Bahia, Brazil, characterizing their gardeners in terms of socioeconomic aspects, and to systematize information about the food produced and the use of NUS.

## Methods

A cross-sectional, quantitative study was carried out throughout urban gardens located in the urban area of the municipality of Salvador, Brazil. The study included two steps: (i) mapping of existing urban gardens; (ii) site visits and data collection, with the application of a semi-structured questionnaire and verification of food grown, destination of production, availability, and use of NUS in urban gardens. Fieldwork was conducted between September 2018 and May 2019.

The municipality of Salvador is located on the coast of the state of Bahia (between the geographical coordinates 12° 59′ 36.0″ South and 38° 31′ 16.0″ West of Greenwich), constituting the economic, political, and administrative center of the state. The city is the fourth largest capital in Brazil, occupying an area of 693.453 km^2^. It has an estimated population of 2,886,698 inhabitants and a demographic density of 3859.44 inhabitants/km^2^. As for the political-administrative organization of the municipality, it comprises 10 administrative regions called neighborhood prefectures and 12 sanitary districts [24, 25].

For the gardens’ localization in the urban area of the municipality, initial contacts were made with the Environmental Health Surveillance sector of the Municipal Health Secretariat as well as with community leaders in the sub-municipal political bodies. Information obtained from previous studies with urban gardens in the city was also considered [21–23]. For the geographical location of the gardens, Google Maps was used. In addition, visits to the indicated places were made in order to confirm the existence and functioning of the gardens.

After locating the gardens, site visits were conducted, and the responsible gardeners (manager and main caretaker) were contacted. As inclusion criteria in the study, the gardens that were active, producing vegetables and/or fruits, and who voluntarily agreed to participate in the research were considered. For the study, private gardens were considered those under the management of a single gardener who may have subordinate workers—in general, the production was mainly intended for commercialization. Community gardens were those that had a gardener in charge (leader) and whose division of labor was shared with other members of the community (volunteers)—the production was mostly intended for subsistence and donation, but could also be marketed.

The managers of the gardens (17 gardeners) answered a semi-structured questionnaire, which included information on socioeconomic aspects (age, marital status, proximity to the garden, education level, family income, working in another profession, and receiving financial benefits from the government); main food grown and destination of production (consumption, donation, and selling); and availability and use of NUS (as food and for medical purposes).

During the visits, the research team, composed of a nutritionist, biologist (botanical specialist), and agronomist, proceeded to walk through the gardens in order to verify the availability of NUS, registering, in a form, the species identified (only the plant species recognized by the researchers were recorded). The plants were also photographed in their natural environment. The scientific and popular names of the plants found were confirmed by consulting a Brazilian guide to identify these species [17], which was developed by researchers of the field and published by Instituto Plantarum—a reference center for research and conservation of Brazilian flora.

This publication is an illustrated identification guide, which provides detailed photographs of these plants, descriptions of their morphological characteristics, and other information such as origin and natural habitat, ways of propagation, parts utilized, and culinary uses. The vouchers of the plants cataloged in this guide are deposited at the Herbarium of the Botanical Garden Plantarum (HPL), in Nova Odessa, São Paulo, Brazil and at the Herbarium of the Federal Institute of Education, Science and Technology of Amazonas (EAFM), in Manaus, Amazonas, Brazil. Finally, the names of the plant species were checked and updated through consultation with Flora do Brasil 2020 - Botanical Garden, an online system of information on species of the Brazilian flora [26].

Data were tabulated and analyzed by descriptive statistics using the software IBM SPSS version 20 for Windows. Association tests (chi-square) were also applied in order to investigate possible relations between respondents’ socioeconomic characteristics, the availability of NUS in the gardens, and consumption by the families. The probability level adopted in the test was 0.05.

The research project was approved by the Research Ethics Committee of the Federal University of Bahia School of Nutrition (Opinion No. 2.848.192). All research participants signed the informed consent form (ICF), expressing their consent to the conditions of the study and authorizing photographic registration of the plants in their natural place of occurrence.

## Results and discussion

### Mapping of the urban gardens

**Fig. 1:**
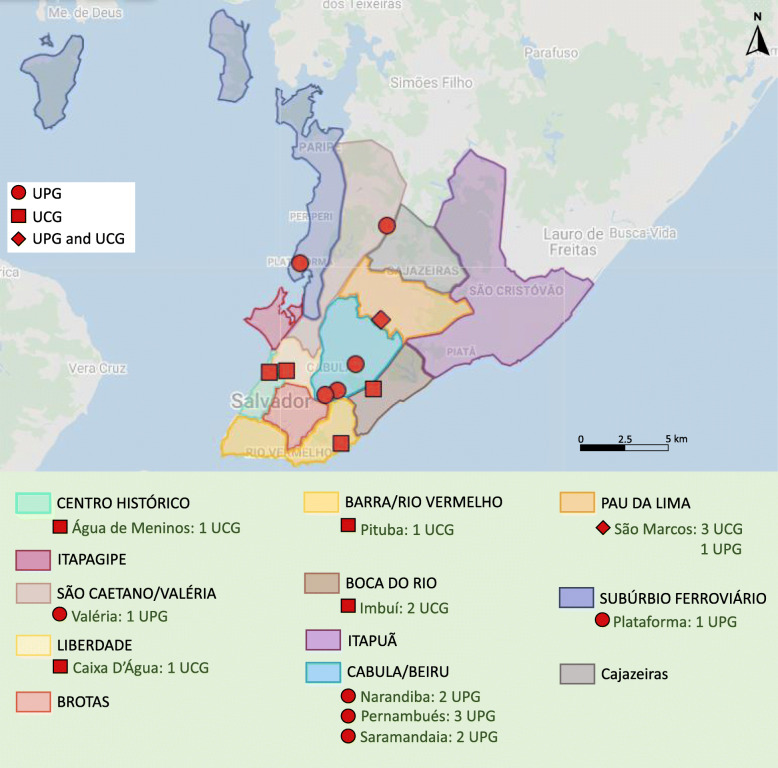
Spatial distribution of sanitary districts, neighborhoods, and active urban gardens in Salvador, Brazil, 2019. Adapted from Google Maps 2020 https://www.google.com/maps

Initially, the information collected indicated the existence of 27 active urban gardens, 10 communal (UCG) and 17 private (UPG), distributed in 17 neighborhoods. Considering Salvador’s organization in sanitary districts, urban gardens were established in nine of the 12 existing districts. However, it was found that 17 of these gardens were active and with production. In addition, during the visits, one more urban garden was located. Thus, it was possible to identify 18 active gardens (8 communal and 10 private), with vegetable and/or fruit production, in the municipality (Fig. 1).

**Fig. 2:**
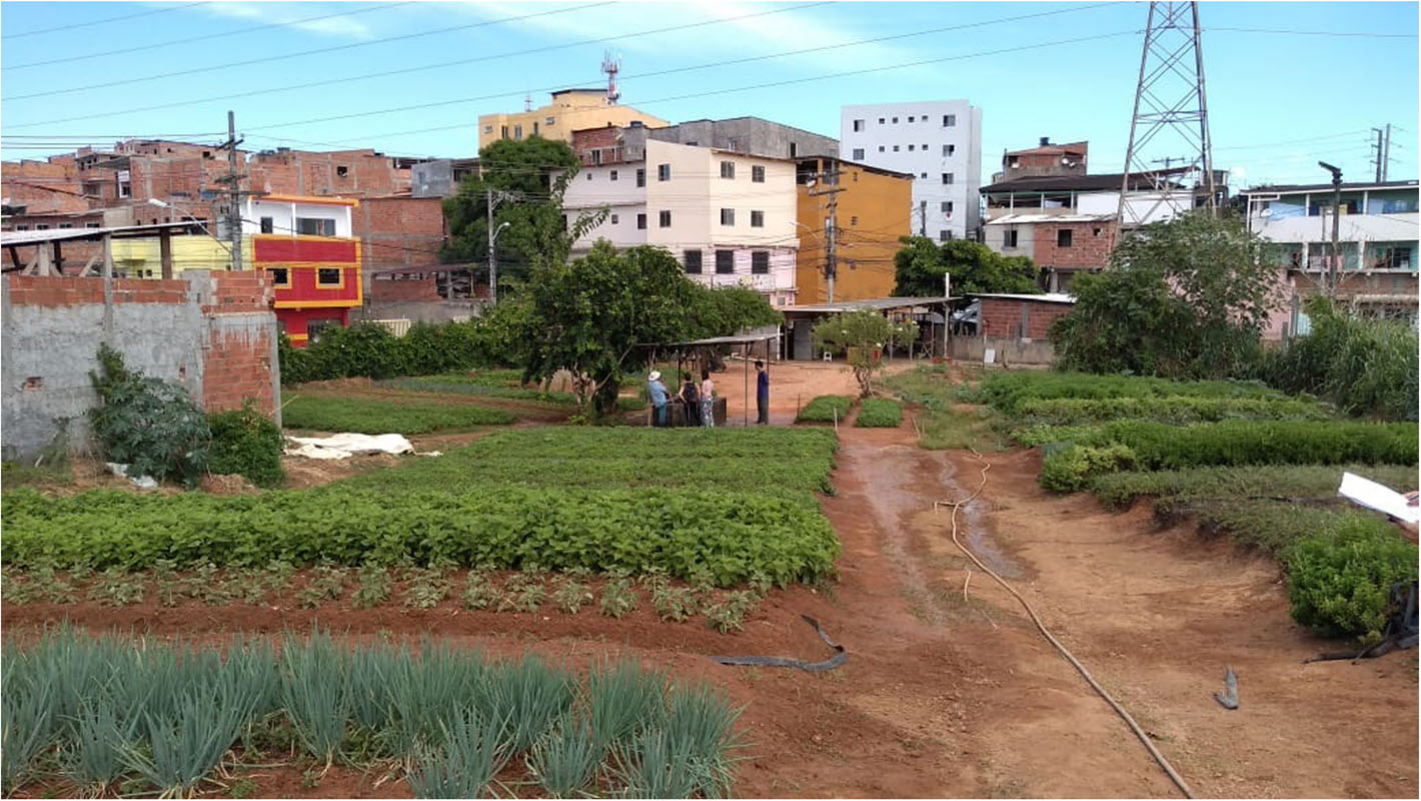
An urban garden identified in the research in Salvador, Brazil, 2019. Photography from the authors

Through the visits to the gardens, it was possible to observe the way in which they are distributed around the urban area of the city (Fig. 2). Since the gardener in charge of one private garden was not found, even after trying to approach him a second time, 17 gardens (8 communal and 9 private) were included in the next stages of the research.

Based on the number of urban gardens identified, there has been a reduction in their quantity in Salvador, Brazil, over the years. In 2013, there were 49 urban gardens in the municipality [21], compared with 18 active urban gardens with food production in 2019—a reduction of 63.3% in 6 years.

During the visits to the gardens, it was found that some of them, located in public areas, no longer existed, with the area being used for the construction of squares and highways. Although research in different countries has shown that green spaces in residential areas can contribute to food security and the sustainability of urban systems [27–29] and the lack of government support, as well as the absence of public policies aimed at supply and urban agriculture, can contribute to the decline in municipal agricultural production [5], as also observed in the present study.

Most Brazilian municipalities have no legal framework or legislation that promotes the strengthening of urban agriculture [8, 23], as is the case of Salvador. However, supporting efforts in order to recover and sustain food production sources are important [30, 31]. In Belo Horizonte, Minas Gerais state, the establishment of the Urban Horticulture Support Policy, in which the development of agriculture is guided by a Food Security Council, recognizes the contribution of the activity to the development of the social functions of the municipality [5]. The city of Curitiba, Paraná state, has approved a bill authorizing the occupation of public and private spaces for the development of urban agriculture, seeking to promote sustainable urban production and improve the food security of the population [32].

Cities, when well-managed, can be sources of solutions to the challenges of urbanization [33]. In European cities, in Italy and France, for example, urban gardens are increasingly popular initiatives [29, 34]. In Paris, France, urban gardens are set up on abandoned land and are managed by associations, which sign a contract with local authorities or landowners, specifying the activities to be carried out and the association’s duties, including favoring environmentally sound practices. The initiatives also have the support of the municipality, which also participates in the contract, assuming some responsibilities, such as ensuring the supply of adequate equipment and soil, as well as water supply [29, 35].

#### Socioeconomic aspects of the gardeners

Regarding the socioeconomic profile of gardeners responsible for the gardens (Table 1), in Salvador, Brazil, it was found that urban agriculture was mainly developed by older people over the age of fifty, some of them elderly (41.2%) most of whom were married. The vast majority of respondents (with the exception of one gardener) lived close to gardens, which facilitated the access and conduction of activities, especially for the older ones.

**Table 1:** Socioeconomic profile of gardeners in charge of urban gardens in Salvador, Brazil, 2019

Indicator	Distribution		
	UCG	UPG	Overall
**Age (years)**			
Range	31–67	51–66	31–67
Average (standard deviation)	52.50 (11.39)	58.67 (5.43)	55.76 (9.03)
	% (*n*)	% (*n*)	% (*n*)
**Sex**			
Female	87.5 (7)	11.1 (1)	47.1 (8)
Male	12.5 (1)	88.9 (8)	52.9 (9)
**Marital status**			
Single	37.5 (3)	22.2 (2)	29.4 (5)
Married	37.5 (3)	44.4 (4)	41.2 (7)
Divorced	25.0 (2)	11.1 (1)	5.9 (1)
Separated	–	11.1 (1)	17.6 (3)
Widowed	–	–	–
Civil partnership	–	11.1 (1)	5.9 (1)
**Resides near garden**			
Yes	87.5 (7)	100 (9)	94.1 (16)
No	12.5 (1)	–	5.9 (1)
**Schooling**			
Illiterate	–	22.2 (2)	11.8 (2)
Unfinished elementary school	12.5 (1)	77.8 (7)	47.1 (8)
Finished elementary school	–	–	–
Unfinished high school	–	–	–
Finished high school	25.0 (2)	–	11.8 (2)
Unfinished university	12.5 (1)	–	5.9 (1)
Finished university	50.0 (4)	–	23.5 (4)
**Family monthly income**^a^			
Less than a minimum wage	14.3 (1)	11.1 (1)	12.5 (2)
One to two minimum wages	28.6 (2)	77.8 (7)	56.3 (9)
Two to three minimum wages	–	–	–
Three to four minimum wages	14.3 (1)	11.1 (1)	12.5 (2)
Four to five minimum wages	14.3 (1)	–	6.3 (1)
Over five minimum wages	28.6 (2)	–	12.5 (2)
**Other profession**			
Yes	50.0 (4)	22.2 (2)	35.3 (6)
No	50.0 (4)	77.8 (7)	64.7 (11)
**Garden is main source of income**			
Yes	12.5 (1)	88.9 (8)	52.9 (9)
No	25.0 (2)	11.1 (1)	17.6 (3)
Not applicable	62.5 (5)	–	29.4 (5)
**Financial government benefits**^a^			
Yes	57.1 (4)	33.3 (3)	43.8 (7)
No	42.9 (3)	66.7 (6)	56.3 (9)

^a^It was possible to consider only seven UCG

Most of the interviewees were male (mainly in UPG), had a low level of formal education (up to incomplete elementary school), and monthly family income of a maximum of three to four minimum wages. In the UCG, female gardeners predominated, with higher schooling levels and higher monthly family income, reaching more than five minimum wages. However, the majority of UCG respondents had another occupation (merchant, civil servant, traffic engineer, teacher, and researcher) and did not hold the garden as a source of income, carrying out activities on a voluntary basis, as a result of a municipal government initiative that has favored the implementation of community gardens in the city’s wealthiest neighborhoods, attracting residents, volunteers, with better socioeconomic conditions [36].

Conversely, most gardeners (seven at UPG and three at UCG) did not have another occupation and most of the interviewees from UPG reported having the gardens as the main source of income, depending on them to guarantee the sustenance of their families. Seven of the respondents said they received retirement or other benefits from the government (Bolsa Família Program), but the minority of them (three gardeners) worked in UPG. In Salvador, less than a third of the total population (29.7%) has a formal occupation [25]; thus, for many families, informal jobs such as the ones available at urban gardens are a way of ensuring income.

It is clear, therefore, that, in the event of the extinction of these gardens (which has already happened with others mentioned in this study), these families may fall into a situation of food insecurity. A recent study by Souza et al. [37], with urban gardens in Salvador, Brazil, serves as an alert to this problem. The research shows little technical and financial support for urban agriculture and signals the need to formulate policies for the segment, in order to promote better organization, strengthening, and continuity of the activity.

While in Salvador, Brazil, the main motivation for the practice of urban agriculture is income generation, in cities in developed countries, like the USA, France, and Italy, the main reasons for carrying out the activities in gardens is the desire for sustainable green spaces in cities, for healthier food, and for personal well-being (like the pleasure generated by contact with nature) [34, 38, 39]. In a study by Ruggeri et al. [34], some socioeconomic features described about horticulturists in Milan, Italy, resemble the observations in the present study, such as the predominance of male gardeners (88.0%), of a more advanced age group (66 years in average). In contrast, the gardeners had a better education level (mostly high school) and a higher monthly income (between 1500 and 2000 euros), when compared with the majority of their counterparts in Salvador, Brazil, and most of them (87.0%) were retired, using their free time to develop activities in the gardens.

It is worth mentioning that in the fields “Monthly Family Income” and “Government Financial Benefits”, all UPG were included, but seven of the eight UCG were considered. It was not possible to apply these questions to one of them, as it was situated in a community that sheltered homeless people, maintained mainly by donations and with high resident turnover. Thus, these requirements were applied to just sixteen gardens in total.

### Cultivated food and destination of production

The gardeners cited a variety of vegetables and fruits commonly grown in the gardens. Lettuce, mint, chives, cilantro, kale, basil, arugula, okra, cassava, tomato, mango, banana, papaya, avocado, passion fruit, acerola cherry, mombin, orange, guava, and coconut were the main foods cited.

Most of the production was for commercialization, in 70.6% of the gardens (three UCG and nine UPG), and donation, in 47.1% of the gardens (five UCG and three UPG), contributing to the municipal food supply. Moreover, food produced in the gardens was consumed by 82.4% of gardeners and their families. Similarly, in other South American cities, such as Quito, Ecuador, and Lima, Peru, urban agriculture production also is destined for commercialization, consumption, and donation, which contributes to the improvement of food security in the families [5].

Most of the urban gardens in Salvador, Brazil, that donated part of their production (mainly to nursing homes and schools) were community-based. However, the study showed that the quantity of these (five gardens) is still low when compared with other Brazilian cities, such as Belo Horizonte, Minas Gerais, which has 48 community gardens [5], and Teresina, Piauí, with 42 urban gardens [40]. In developed countries, this number may be much higher. In Madison, Wisconsin, USA, about 33.0% of the households participate in community garden activities, and it is estimated that there are 45,193 of these gardens in the county, providing fresh fruits and vegetables to food insecure populations [27].

In developing countries, urban agriculture can play an important role in FNS, as has been the case in some African countries [41]. In Zimbabwe, 70% of the population is below the poverty line and, due to the worsening economic situation in the country and food insecurity, the population has adopted different survival strategies, including the intensification of urban agriculture, which has generated a positive impact on families [42]. In the current socio-political and economic scenario in Brazil, where the problem of hunger is once again a matter of concern, the intensification of agricultural practice by urban families could be, as in African countries, a relevant strategy for achieving the human right to healthy eating [43].

### Availability and use of NUS

**Fig. 3:**
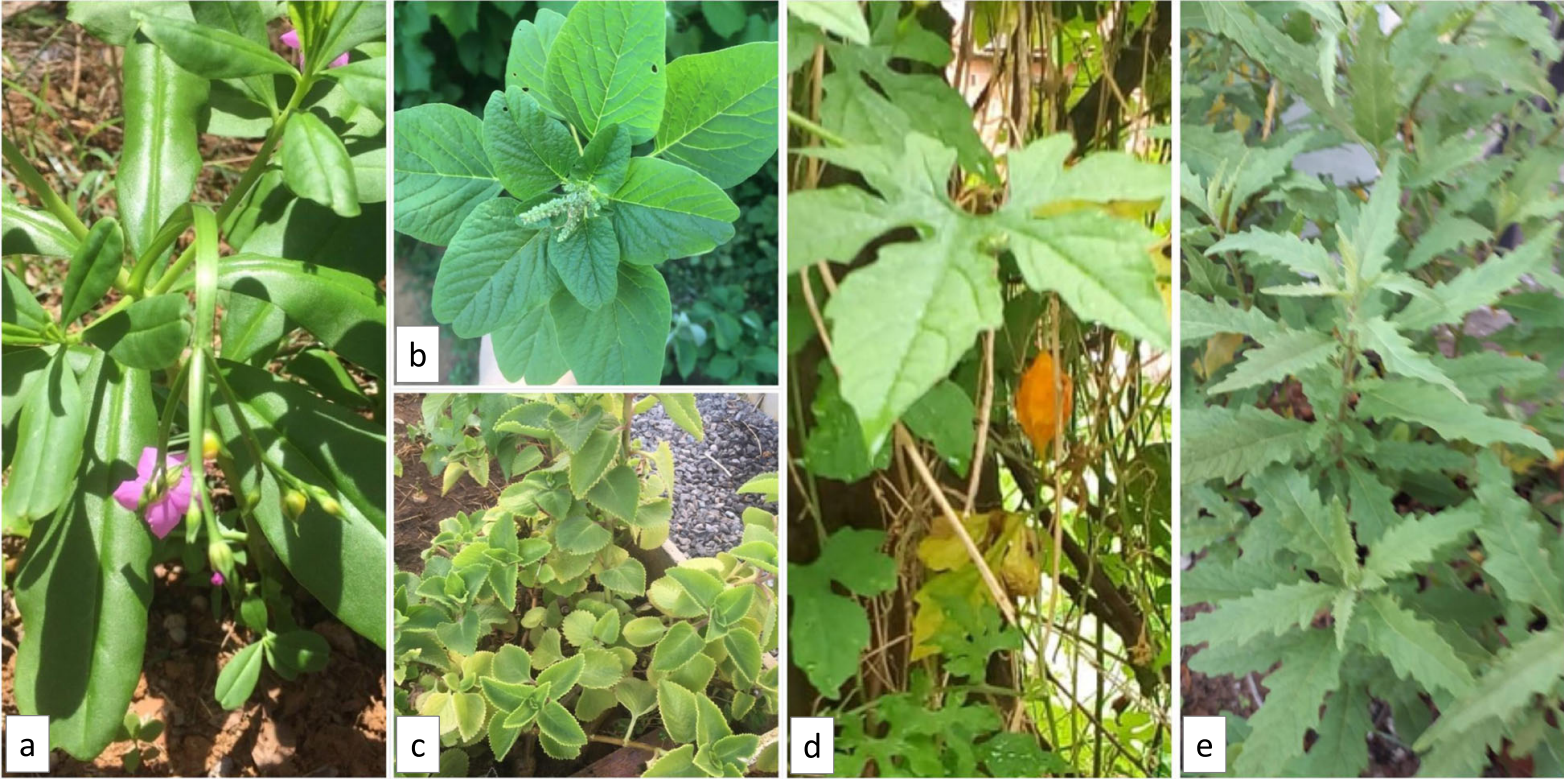
NUS photographed in urban gardens in Salvador, Brazil, 2019. **a** *Talinum fruticosum* (L.) Juss. **b** *Amaranthus deflexus* L. **c** *Coleus amboinicus* Lour. **d** *Momordica charantia* L. **e** *Dysphania ambrosioides* (L.) Mosyakin & Clemants. Photography from the authors

Among the species of plants grown in urban gardens, few NUS were cited. Nonetheless, during the walk through the gardens, to verify the availability of the plants, a total of fifty-nine NUS were found (Table 2). Images of five species of greater occurrence, photographed in their natural environment, can be seen (Fig. 3).

**Table 2:** NUS in descending order of occurrence in the gardens of Salvador, Brazil, 2019

Scientific name	Common names in Brazil^a^	Occurrence		
		UCG	UPG	Overall
		% (*n*)	% (*n*)	% (*n*)
*Talinum fruticosum* (L.) Juss.	língua-de-vaca; cariru; beldroega-graúda	100.0 (8)	77.8 (7)	88.2 (15)
*Amaranthus deflexus* L.	caruru; caruru-rasteiro; bredo	50.0 (4)	100.0 (9)	76.5 (13)
*Coleus amboinicus* Lour.	hortelã-grosso; hortelã-graúda; hortelã-da-bahia	100.0 (8)	55.6 (5)	76.5 (13)
*Momordica charantia* L.	melão-de-são-caetano; goya; melãozinho	50.0 (4)	88.9 (8)	70.6 (12)
*Dysphania ambrosioides* (L.) Mosyakin & Clemants	mastruz; erva-de-santa-maria; lombrigueira	50.0 (4)	66.7 (6)	58.8 (10)
*Portulaca oleracea* L.	beldroega; caaponga; porcelana	62.5 (5)	55.6 (5)	58.8 (10)
*Eryngium foetidum* L.	coentro-bravo; coentro-da-índia; coentro-de-caboclo	37.5 (3)	66.7 (6)	52.9 (9)
*Schinus terebinthifolia* Raddi	aroeira; pimenta-rosa; aguaraíba	62.5 (5)	33.3 (3)	47.1 (8)
*Xanthosoma taioba* E. G. Gonç.	taioba; inhame-de-folha; taiá	62.5 (5)	33.3 (3)	47.1 (8)
*Tripogandra diuretica* (Mart.) Handlos	trapoeraba; trapuerava; ondas-do-mar	50.0 (4)	44.4 (4)	47.1 (8)
*Pereskia aculeata* Mill.	ora-pro-nóbis; carne-de-pobre; lobrobó	75.0 (6)	11.1 (1)	41.2 (7)
*Turnera subulata* Sm.	chanana; albina; flor-do-guarujá	75.0 (6)	11.1 (1)	41.2 (7)
*Cymbopogon citratus* (DC.) Stapf	capim-santo; erva-cidreira; capim-limão	62.5 (5)	22.2 (2)	41.2 (7)
*Cajanus cajan* (L.) Huth	andu; guandu; ervilha-do-congo	87.5 (7)	–	41.2 (7)
*Peperomia pellucida* (L.) Kunth	peperômia; erva-de-jabuti; alfavaca-de-cobra	25.0 (2)	44.4 (4)	35.3 (6)
*Ocimum campechianum* Mill.	alfavaquinha; alfavaca-de-galinha; afavaca-do-mato	50.0 (4)	22.2 (2)	35.3 (6)
*Cucumis anguria* L.	maxixe; maxixo; pepino-espinhoso	50.0 (4)	11.1 (1)	29.4 (5)
*Moringa oleifera* Lam.	moringa; quiabo-de-quina	50.0 (4)	–	23.5 (4)
*Solanum stramoniifolium* Jacq.	jurubeba; jurubeba-vermelha; jurubeba-do-roçado	25.0 (2)	22.2 (2)	23.5 (4)
*Sonchus oleraceus* L.	serralha; serralheira; chicória-brava	37.5 (3)	11.1 (1)	23.5 (4)
*Basella alba* L.	bertalha; couve-mimosa; espinafre-de-malabar	25.0 (2)	11.1 (1)	17.6 (3)
*Plantago major* L.	tansagem; tanchagem; plantagem	25.0 (2)	11.1 (1)	17.6 (3)
*Trichosanthes cucumerina* L.	quiabo-de-metro; cabaça-cobra; abóbora-jiboia	12.5 (1)	22.2 (2)	17.6 (3)
*Curcuma longa* L.	açafrão-da-terra; açafrão-da-índia; cúrcuma	37.5 (3)	–	17.6 (3)
*Solanum americanum* Mill.	maria-pretinha; erva-moura; caraxixá	12.5 (1)	22.2 (2)	17.6 (3)
*Ocimum gratissimum* L.	alfavaca-cravo; alfavacão; quioiô	25.0 (2)	11.1 (1)	17.6 (3)
*Mentha pulegium* L.	poejo; hortelãzinho	25.0 (2)	–	11.8 (2)
*Hibiscus rosa-sinensis* L.	hibisco; graxa-de-estudante; mimo-de-vênus	12.5 (1)	11.1 (1)	11.8 (2)
*Cnidoscolus aconitifolius* (Mill.) I. M. Johnst.	chaya; espinafre-selvagem; urtiga-branca	25.0 (2)	–	11.8 (2)
*Piper umbellatum* L.	capeba; capeva; aguaxima	12.5 (1)	11.1 (1)	11.8 (2)
*Boerhavia diffusa* L.	pega-pinto; erva-tostão; tangará	12.5 (1)	11.1 (1)	11.8 (2)
*Oxalis barrelieri* L.	azedinha; trevo-arbustivo; trevo-amazônico	12.5 (1)	11.1 (1)	11.8 (2)
*Boehmeria caudata* Sw.	urtiga-mansa; assa-peixe; folha-de-santana	12.5 (1)	11.1 (1)	11.8 (2)
*Averrhoa bilimbi* L.	biri-biri; limão-caieno; bilimbi	25.0 (2)	–	11.8 (2)
*Costus amazonicus* (Loes.) J. F. Macbr.	cana-de-macaco; cana-do-mato; pobre-velho	12.5 (1)	11.1 (1)	11.8 (2)
*Bidens pilosa* L.	picão-preto; pico-pico; carrapicho-de-agulha	–	22.2 (2)	11.8 (2)
*Brassica juncea* (L.) Czern.	mostarda; mostarda-verde; mostarda-chinesa	12.5 (1)	11.1 (1)	11.8 (2)
*Solanum betaceum* Cav.	tomatinho-do-mato; tomate-francês; tamarilho;	25.0 (2)	–	11.8 (2)
*Spondias purpurea* L.	seriguela; cirigueleira; ciriguela	25.0 (2)	–	11.8 (2)
*Genipa americana* L.	jenipapo; jenipapeiro; jenipapinho	12.5 (1)	11.1 (1)	11.8 (2)
*Canavalia ensiformis* (L.) DC.	feijão-de-porco; feijão-espada	12.5 (1)	–	5.9 (1)
*Galinsoga parviflora* Cav.	picão-branco; fazendeiro; brinco-de-princesa	12.5 (1)	–	5.9 (1)
*Fridericia chica* (Bonpl.) L. G. Lohmann	crajiru; chica; cipó-cruz	12.5 (1)	–	5.9 (1)
*Echinodorus macrophyllus* (Kunth) Micheli	chapéu-de-couro; chá-de-campanha; erva-do-brejo	12.5 (1)	-	5.9 (1)
*Piper peltatum* L.	santa-maria; caapeba-amazônica; folha-de-arraia	12.5 (1)	–	5.9 (1)
*Physalis pubescens* L.	fisális; joá-de-capote; balãozinho	12.5 (1)	–	5.9 (1)
*Kalanchoe pinnata* (Lam.) Pers.	folha-da-fortuna; corama; pirarucu	12.5 (1)	–	5.9 (1)
*Conyza bonariensis* (L.) Cronquist	buva; erva-lanceta; voadeira	12.5 (1)	–	5.9 (1)
*Terminalia catappa* L.	castanhola; sete-copas; chapéu-de-sol	–	11.1 (1)	5.9 (1)
*Pteridium aquilinum* (L.) Kuhn	samambaia; samambaia-das-taperas; feto	–	11.1 (1)	5.9 (1)
*Artocarpus altilis* (Parkinson) Fosberg	fruta-pão; fruta-pão-de-massa	–	11.1 (1)	5.9 (1)
*Pouteria caimito* (Ruiz & Pav.) Radlk.	abiu; caimito; guapeva	–	11.1 (1)	5.9 (1)
*Celosia argentea* L*.*	celosia; espinafre-africano; crista-plumosa	12.5 (1)	–	5.9 (1)
*Maranta arundinacea* L.	araruta; raruta; maranta	12.5 (1)	–	5.9 (1)
*Vigna unguiculata* (L.) Walp.	feijão-de-corda; feijão-de-praia; feijão-fradinho	12.5 (1)	–	5.9 (1)
*Sicana odorifera* (Vell.) Naudin	melão-croá; melão-caboclo; maracujina	12.5 (1)	–	5.9 (1)
*Asystasia gangetica* (L.) T. Anderson	espinafre-da-índia; coromandel; violeta-chinesa	12.5 (1)	–	5.9 (1)
*Hylocereus undatus* (Haw.) Britton & Rose	pitaia; dama-da-noite; pitaia-branca	12.5 (1)	–	5.9 (1)
*Syzygium malaccense* (L.) Merr. & L.M. Perry	jambo; jambo-vermelho; jambo-roxo	–	11.1 (1)	5.9 (1)

^a^Up to three common names were presented for each plant

Neglected and underutilized plant species are described worldwide and their variety is remarkable: 539 vegetables and 645 fruits in Africa, 2800 fruits in the tropics, 200 leafy vegetables in Kenya, 228 vegetables in Southeast Asia, and 137 native vegetables in Italy [44]. In Brazil, there is a wide variety of NUS, with over 3000 species recorded in the literature [17, 45–47].

Studies show that the NUS found in urban gardens in Salvador have the potential for complementing food, perpetuating healthy eating practices, and promoting adequate nutrition. Research with different species of these plants has revealed that they are rich in nutrients, such as proteins, fibers, vitamins, minerals, essential oils, and bioactive compounds with antioxidant capacity, which justifies the stimulus for consumption [17, 48–56].

Although some species of plants found in urban gardens—*Coleus amboinicus* Lour., *Dysphania ambrosioides* (L.) Mosyakin & Clemants, *Schinus terebinthifolia* Raddi, *Cymbopogon citratus* (DC.) Stapf, *Cucumis anguria* L., *Curcuma longa* L., *Spondias purpurea* L., *Genipa americana* L., *Eryngium foetidum L.*—may be considered NUS in the country, they are traditionally consumed by the population of Bahia, Brazil. Nonetheless, as with most NUS, they are not widely commercialized.

**Fig. 4:**
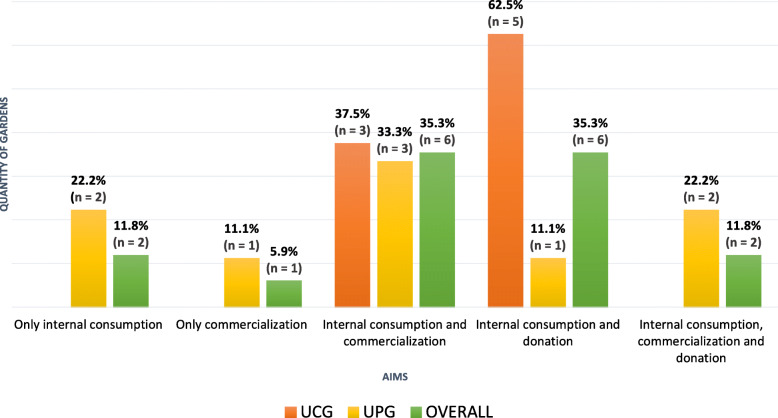
Aims of NUS grown in the urban gardens of Salvador, Brazil, 2019

In most community urban gardens studied (five of them), NUS were intended for internal consumption and donation. In contrast, most of the UPG (six gardens) traded NUS (Fig. 4). In this context, three species of NUS—*Coleus amboinicus* Lour., *Eryngium foetidum* L. and *Cucumis anguria* L.—were cultivated, since they are part of the Bahian population’s food culture, thus commercialized in the gardens, fairs, or small markets.

Most NUS that grew spontaneously were often uprooted by gardeners, mainly because they had no important commercial value and/or were considered by them as “weeds.” The monoculture of a limited number of vegetables and the competitiveness in modern agriculture has been causing the disappearance of NUS, which lose space in the global trading circuit, surviving only in small local markets [44]. Besides having potential to improve FNS, cultivation of these plants could promote greater genetic biodiversity, contributing to the maintenance of the ecosystem [13, 57].

**Fig. 5:**
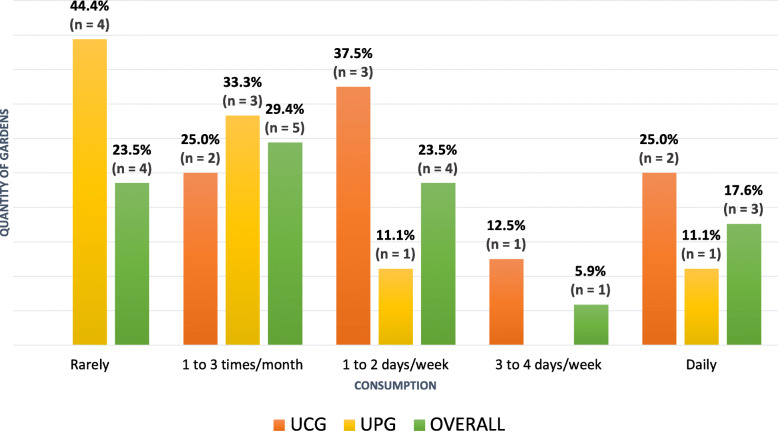
Frequency of consumption (%) of NUS by the gardeners in Salvador, Brazil, 2019

Concerning the frequency of NUS consumption by gardeners and their families (Fig. 5), four respondents in charge of UPG reported that their consumption was rare. Although seldom cultivated and spontaneously grown, 76.5% (all UCG and five UPG respondents) of the gardeners declared using NUS in their family food.

**Fig. 6:**
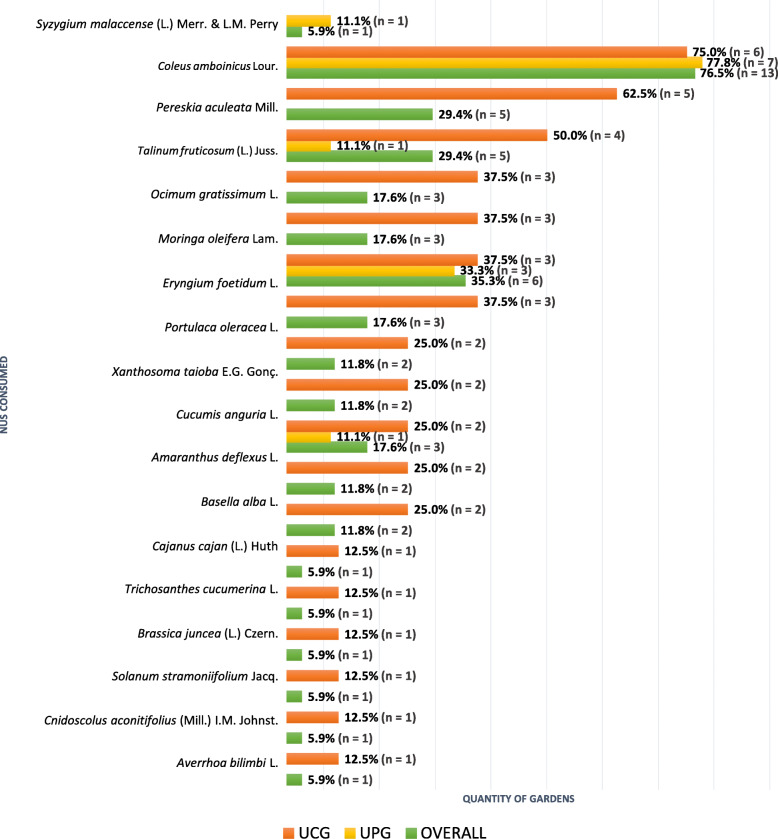
NUS consumed by urban gardeners in Salvador, Brazil, 2019

Gardeners reported utilizing nineteen types of NUS to feed their families (Fig. 6).

UPG gardeners (mostly men) reported consuming five species of NUS, while eighteen species of plants were cited by UCG interviewees (women, mostly), which represents 30.5% of the total NUS found in urban gardens. This finding agrees with the analysis of Padulosi [44], for whom women have been playing an important role in the use of different NUS, promoting the interaction between agrobiodiversity and nutritional security of their families.

Most respondents (70.5%) also reported using some of the available NUS for medical purposes, including *Dysphania ambrosioides* (L.) Mosyakin & Clemants, *Plantago major* L., *Schinus terebinthifolia* Raddi, *Coleus amboinicus* Lour., *Moringa oleifera* Lam., *Solanum stramoniifolium* Jacq., *Cymbopogon citratus* (DC.) Stapf, *Eryngium foetidum* L., *Ocimum gratissimum* L., and *Solanum americanum* Mill. The female respondents also reported using a greater amount of NUS for medicinal purposes, nine of the aforementioned species, while men reported using six of them. Silva et al. [58] carried out a survey on plants used as medicines in rural communities in Piauí, Brazil, and found that women had a fundamental role in the cultivation and use of these vegetables, as well as in the maintenance of having a more specific knowledge of herbs and shrubs used in homemade therapy.

In the present study, it was possible to observe a greater occurrence and a greater use of NUS in UCG, whose respondents were mainly female and had a higher level of education, demonstrating a certain knowledge about the importance of NUS. However, statistical tests performed to verify association between these variables showed no association between occurrence and use of NUS with the type of garden (private or community), nor between the occurrence and use of NUS with the sex or education of gardeners.

As for the main forms of consumption of NUS by the respondents’ families (Table 3), the most utilized parts of plants were leaves (68.4%), with fruits (26.3%) and seeds (5.3%) in smaller proportion. The species are mostly eaten raw, cooked, or sautéed in various preparations, in the form of juices or used as condiments. In recent years, an increasing number of chefs have been using NUS in their dishes and many have become “gourmet food,” mostly eaten by people with higher purchasing power [14, 44]. However, NUS can contribute to the diversification of menus of families of all socioeconomic classes. In the present study, a large availability of NUS was found in urban gardens, observing its use in various culinary preparations. This finding confirms the great potential these plants have for food and can be used in a wide variety of dishes.

**Table 3:** Forms of NUS consumption by gardeners from urban gardens of Salvador, Brazil, 2019

Plant	Consumed part	Ways to prepare	Preparations
**Vegetable/seasoning**^a^			
*Amaranthus deflexus* L.	Leaf	Cooked and sautéed	Cooked with other NUS; cooked with beans or meat; sautéed with condiments
*Averrhoa bilimbi* L.	Leaf	Condiment	Condiment for chicken preparations
*Basella alba* L.	Leaf	Raw, cooked and sautéed	Raw and/or cooked salads; omelets; salty pies; soups; sautéed rice; braised with condiments
*Brassica juncea* (L.) Czern	Leaf	Sautéed	Sautéed with seasonings (similar to the preparation of cauliflower); sautéed and added in the preparation of *farofa*
*Cucumis anguria* L.	Fruit	Cooked and sautéed	Cooked with meat or chicken; Sautéed with condiments
*Cnidoscolus aconitifolius* (Mill.) I.M. Johnst.	Leaf	Sautéed	Sautéed with seasonings (similar to the preparation of cauliflower); sautéed and added in the preparation of *farofa*
*Eryngium foetidum* L.	Leaf	Condiment	Condiment for the preparation of fish in general and for *moqueca*
*Moringa oleifera* Lam.	Leaf	Raw, cooked and juice	Raw salad; preparation for cakes and breads; juices and shakes.
*Ocimum gratissimum* L.	Leaf	Cooked and as condiment	Cooked with beans; seasoning for the preparation of various recipes with chicken and meat
*Pereskia aculeata* Mill.	Leaf	Raw, cooked and sautéed	Raw salads; omelets; cooked with chicken; salty pies; pâté; sautéed rice; braised with condiments
*Coleus amboinicus* Lour.	Leaf	Raw, juice and as condiment	Raw salads; green juice; seasoning for the preparation of meat, fish, and chicken in general and for various preparations such as boiled chicken and beans
*Portulaca oleracea* L.	Leaf	Raw and cooked	Raw salads; cooked with beans or chicken.
*Schinus terebinthifolia* Raddi	Fruit	Condiment	Spice for various dishes (similar to black pepper)
*Solanum stramoniifolium* Jacq.	Fruit	Raw	Raw Salads
*Talinum fruticosum* (L.) Juss.	Leaf	Cooked and sautéed	Cooked with beans or shrimp and/or pepperoni sausage; sautéed with seasonings; braised with eggs or shrimps
*Trichosanthes cucumerina* L.	Fruit	Raw, cooked and sautéed	Raw salads; cooked or sautéed with condiments
*Xanthosoma taioba* E.G. Gonç.	Leaf	Cooked and sautéed	As an ingredient in stew; braised with condiments
**Legumes**^a^			
*Cajanus cajan* (L.) Huth	Seed	Cooked	Cooked with spices and sometimes with meat (similar to basic bean preparations)
**Fruit**^a^			
*Syzygium malaccense* (L.) Merr. & L.M. Perry	Fruit	Raw	Consumed *in natura*, as fruit

^a^Food category

A similar study investigated the residents’ knowledge about NUS in Ribeirão da Ilha, Florianópolis, Santa Catarina state, Brazil [59]. In the research, 63 types of NUS were mentioned and the fruit was the most utilized part, followed by seed, root, leaf, flower, and stalk. The most cited forms of consumption were raw, in juices, alcoholic beverages, sauces, sweets, salads, crumbs, and as food coloring. Other studies that sought to investigate the use of NUS in the feeding of local populations have been carried out in some countries, registering different forms of consumption for these vegetables [12, 60–64].

When asked how they learned to use NUS in their preparations, gardeners reported learning mainly from their parents and/or grandparents, but also from neighbors, teachers, and the internet. Respondents were also asked if the youngest members of their families (children, nephews, grandchildren, etc.) were interested in NUS and 76.5% said that the youth showed no interest. However, it is not truly possible to valorize the unknown. Learning about the available NUS is essential for the appreciation and conscious use of species. In this sense, the dissemination of scientific knowledge about NUS and its health benefits must be increasingly encouraged [65–67].

Based on the above, the population’s food literacy could be considered a strategy to connect individuals, families, and communities to urban gardens, in order to promote a healthy relationship with the available food and engagement in a sustainable food system [68–71]. Research shows that the promotion of food literacy in adults and, most importantly, in children and adolescents, when inserted in the school education process, can generate good results, producing immediate and long-term benefits [71–74].

Although studies that investigate the contributions of NUS to the population’s diets are still scarce, research indicates that the consumption of these plants can show contributions. Among them, the reduction of nutritional deficiencies and the offer of local, sustainable, and culturally acceptable solutions to malnutrition problems [14, 16, 75].

In Brazil, 26.5% of the population lives below the poverty line, and in Bahia, this percentage is above the national average, reaching 44.8% of the population (6.9 million people). It is a population that is more exposed to disadvantages associated with poverty, such as restrictions on access to services and rights (e.g., basic sanitation and adequate housing) and food insecurity [76]. To face these problems, it is necessary to establish a movement against hegemony, with a focus on promoting agrobiodiversity, and NUS are one of the keys to this. In this sense, the diversification of production systems, with the insertion of NUS, can improve the health of agroecosystems, contribute to protecting food systems, in addition to supporting family farming and promoting food security [44, 75].

Therefore, incorporating NUS into national nutrition and FNS policies is an important strategy. Brazilian policies and programs already include the promotion of NUS in dietary guidelines, supporting the production and purchase of these plants for inclusion in school feeding [14, 44]. Interministerial Ordinance N° 284/2018, for example, establishes a list of several species of plants of the Brazilian socio-biodiversity, for commercialization purposes, within the scope of operations carried out by the Food Acquisition Program (PAA) [77].

As a challenge, however, most of the urban gardens included in the present research did not work legally. The majority of them (76.5%) was implanted in a public area, without legal possession of the cultivated spaces and none of the respondents was registered as a family farmer, which represents an impediment for supplying food programs such as PAA. In this sense, there are evident gaps in the perspective of public policies aimed at urban agriculture, signaling the need for civil action, decision-making by managers, and investments.

## Conclusions

The study aimed to systematize information about urban gardens, its producers, and the occurrence of NUS in the city of Salvador, Bahia, Brazil, in order to discuss questions regarding supply, healthier eating practices, and sustainability, in line with the national food and nutrition security guidelines.

Based on the gardens’ location survey, it was possible to evidence the lack of records in the competent public agencies about urban agriculture in the municipality, which resulted in greater time spent to set the study into operation. A limited number of gardens compared with previous records were identified, which may reflect the lack of government support and incentive to urban agriculture in the municipality. Nevertheless, it was observed that urban, private, and community gardens have contributed to the city’s food supply, playing an important role in the population’s food and nutrition security and in building a more sustainable urban environment.

Many NUS were available in the city’s urban gardens. Most species grew spontaneously and, especially in private gardens, were removed as “weeds,” favoring the production of high demand foods in the commercialization circuits.

On the other hand, many of the interviewed gardeners, mainly women, have been using some of these vegetables to feed their families. NUS leaves, fruits, and seeds have been cited as ingredients or even as the main element of a large number of culinary preparations, which shows the versatility of the use of these vegetables in the urban population’s diet.

In view of the findings, it has been assessed that the formulation of public policies aimed at urban agriculture is essential to support and promote agricultural activities in the municipality of Salvador, Bahia, Brazil, including strategies for the insertion of NUS in the agricultural production and food for the population. In this sense, the integration between Agrobiodiversity and FNS can bring great benefits to the communities of the municipality and to the environment.

In addition, further research is suggested to investigate the nutritional value of NUS, their potential use for menu diversification, strategies for promoting consumption, and their contribution to the health of individuals. Furthermore, there is a need to encourage studies that can strengthen and accompany the development of urban agriculture, in places where the activity is fragile, in view of the local food system.

## Data Availability

The datasets used and/or analyzed during the current study are available from the corresponding author on reasonable request.

## Abbreviations

NUS: Neglected and underutilized species
PANC: Plantas Alimentícias Não Convencionais
FNS: Food and Nutrition Security
UPG: Urban private gardens
UCG: Urban community gardens
HPL: Herbário do Jardim Botânico Plantarum
EAFM: Herbário do Instituto Federal de Educação, Ciência e Tecnologia do Amazonas
PAA: Programa de Aquisição de Alimentos
